# How and Why Affective and Reactive Virtual Agents Will Bring New Insights on Social Cognitive Disorders in Schizophrenia? An Illustration with a Virtual Card Game Paradigm

**DOI:** 10.3389/fnhum.2015.00133

**Published:** 2015-03-30

**Authors:** Ali Oker, Elise Prigent, Matthieu Courgeon, Victoria Eyharabide, Mathieu Urbach, Nadine Bazin, Michel-Ange Amorim, Christine Passerieux, Jean-Claude Martin, Eric Brunet-Gouet

**Affiliations:** ^1^HANDIReSP EA4047, Université de Versailles Saint-Quentin, Versailles, France; ^2^CIAMS EA4532, UFR STAPS, Université Paris-Sud, Orsay, France; ^3^UMR6285, LabSTICC, Université Bretagne-Sud, Lorient, France; ^4^STIH EA4509, Université Paris-Sorbonne, Paris, France; ^5^Pôle de Psychiatrie, Centre Hospitalier de Versailles, Versailles, France; ^6^LIMSI UPR3251, Université Paris-Sud, Orsay, France

**Keywords:** virtual agents, schizophrenia, social cognition, theory of mind, facial expression

## Abstract

In recent decades, many studies have shown that schizophrenia is associated with severe social cognitive impairments affecting key components, such as the recognition of emotions, theory of mind, attributional style, and metacognition. Most studies investigated each construct separately, precluding analysis of the interactive and immersive nature of real-life situation. Specialized batteries of tests are under investigation to assess social cognition, which is thought now as a link between neurocognitive disorders and impaired functioning. However, this link accounts for a limited part of the variance of real-life functioning. To fill this gap, advances in virtual reality and affective computing have made it possible to carry out experimental investigations of naturalistic social cognition, in controlled conditions, with good reproducibility. This approach is illustrated with the description of a new paradigm based on an original virtual card game in which subjects interpret emotional displays from a female virtual agent, and decipher her helping intentions. Independent variables concerning emotional expression in terms of valence and intensity were manipulated. We show how several useful dependant variables, ranging from classic experimental psychology data to metacognition or subjective experiences records, may be extracted from a single experiment. Methodological issues about the immersion into a simulated intersubjective situation are considered. The example of this new flexible experimental setting, with regards to the many constructs recognized in social neurosciences, constitutes a rationale for focusing on this potential intermediate link between standardized tests and real-life functioning, and also for using it as an innovative media for cognitive remediation.

## Introduction

There is a long history of reductionism in cognitive science, with the progressive dissection of models of mental processes to explain human behavior. The models are tested with experimental paradigms that exclude confounding factors and focus only on the constructs of interest. This approach has demonstrated the impairment of several cognitive functions in patients suffering from schizophrenia: cognitive disorders affecting attention, memory, and executive functions are among the most commonly reported in patients with this pathological condition (Evans et al., 1997; Cirillo and Seidman, 2003). There have also been clinically relevant attempts to present schizophrenia from the standpoint of a social cognitive disorder (Brüne, 2005; Penn et al., 2008). This point of views generated extensive experimental research and, importantly, influenced clinical thinking in several psychiatric centers and modeled new means of psychological treatments. Nevertheless, many elements are still missing to support a social cognitive theory of psychiatric disorders, and, as we will discuss in the following, methodological advances are hoped for to meet this challenge.

In the first part of this article, we will give a quick overview of the impaired processes, which were identified, from the most elementary, such as social perception, emotional perception, and motor resonance to the most complex, such as mentalizing and empathizing. One practical consequence of this view of schizophrenia has been the continual development of batteries of neuropsychological tests targeting non-social and social aspects of cognition, for use by mental healthcare professionals [for a review see Pinkham et al. (2013)]. These assessments, based on the separate measurements of neuropsychological dimensions, have proved relevant for the prediction of functional outcome (Fett et al., 2011), although they leave unexplained a significant part of the variance.

In the second part of this work, we advocate the added value of considering “naturalistic” social cognition as a domain occulted by the classical approach. We will describe how virtual reality settings may prove useful for investigating social interaction, especially through the use of virtual agents. We will discuss the relationships between social cognitive constructs and those arising from researches on immersion phenomena.

In the third part, we will illustrate these ideas with the proposal of a new paradigm based on a virtual affective agent and show how it may be used with patients. We will argue that such experimental settings will prove useful for assessing the intermediate link between cognition and real-life functioning and give some direction to improve the subjective experience and motivation.

## Part I

### Examination of impairments of social cognition in schizophrenia by a reductionist approach

Social cognition can be considered as an entangled set of information processes relating to the perception of others, the representation of oneself, and interpersonal knowledge in a given social context (Beer and Ochsner, 2006), which allows an individual to adapt his/her behavior to the social environment. Disorders of social behavior are prominent in schizophrenia as noted by Brüne: “the most outstanding characteristic of schizophrenia is the inapt, often bizarre behavior of affected individuals (…) It is almost always the deviant social behavior in schizophrenia that renders patients ‘abnormal”’ (2005, p. 135). For those social cognitive disorders may account for these deviances, Penn and coworkers suggested that several primary domains [theory of mind (ToM), perception of emotion in facial expressions, attributional style] or features (i.e., metacognition) should be considered when assessing social cognition in schizophrenia. Each domain relates to some specific constructs, which have been the object of thorough theorization and experiments.

First, the ability to take the mental states (intentions, beliefs, etc.) of other people into account was extensively studied by developmental psychologists since 1990s (Astington and Gopnik, 1991). ToM has been defined as the ability to represent human mental states and to make inferences about other people’s intentions, beliefs and desires, by acknowledging that others may have a mental state different from our own. ToM has been explored with verbal tasks based on stories (Corcoran and Frith, 2003; Janssen et al., 2003; Van der Cruyssen et al., 2009) and with non-verbal tasks involving sequences of pictures (Sarfati et al., 1999; Brunet et al., 2003; Langdon et al., 2006; Vistoli et al., 2011) or a mixture of stories and pictures [for a review, see Bazin et al. (2007) and Brüne (2005)]. Various factors may modulate the ToM performances of schizophrenia patients and one important debate for designing cognitive remediation techniques concerns the automatic or controlled nature of these cognitive skills (Cohen and German, 2010). For instance, verbal instructions may contribute to the controlled component of ToM (Back and Apperly, 2010), an important finding given the widespread use of verbal instructions to remediate social skill deficits.

Second, it is also widely accepted that patients with schizophrenia and or social developmental disorders (autism, Asperger’s syndrome) display poor discrimination of face identity, emotion, and age (Chambon and Baudouin, 2009). The impairment of emotion recognition in these patients is well documented and has been the object of a number of reviews (Edwards et al., 2002; Kohler et al., 2010). One of the principal difficulties observed relates to fear (Edwards et al., 2001) and disgust (Kohler et al., 2003). According to Kohler et al. (2003), neutral or non-emotional expressions may be wrongly interpreted as emotional cues with a negative bias. An intriguing result was reported by Davis and Gibson (2000) who found that schizophrenic patients had emotion recognition deficits with posed emotional pictures although this pattern of deficit was not replicated with genuine emotion pictures. Besides the complexity of the emotion recognition deficit, evidence of impoverished perceptual strategies is now investigated in schizophrenia. There is a growing body of evidence to suggest that schizophrenic patients have shortened or abnormal oculomotor scan paths when confronted with faces expressing emotions (Loughland et al., 2002; Butler et al., 2009), a finding that may be extended to non-social scenes and reliably discriminate patients from healthy subjects (Benson et al., 2012, p. 722). These findings highlight the importance of intact perceptual strategies for an understanding of the affective states of other people. Other studies have examined the importance of contextual or situational cues for the perception of emotional and mental states in others. For instance, it has been shown that contextual information (e.g., background scenes or body language) can affect the recognition of facial emotions (Aviezer et al., 2009). It has been suggested that the inefficient integration of social contextual information may limit the ability to infer mental and emotional states from facial expressions (Green et al., 2007).

Third, schizophrenic patients have also been reported to have another type of abnormality, relating to attributional style. According to Penn et al. (2008), attributional style concerns the explanations people find for positive and negative events (p. 409) – the way they attribute responsibility, error and merit to others or to themselves. As an argument for the discriminant validity of the attributional style construct, a recent work from Mancuso et al. (2011) showed with a population of 85 schizophrenic outpatients that attributional style measures loaded on a different factor than social perception and mentalizing, and that this factor correlated selectively with positive symptoms, depression/anxiety, and agitation.

Finally, metacognition may be conceptualized in its broadest sense as an individual’s knowledge/representations about his or her own cognitive processes. As explained by Passerieux et al. (2012), “When we answer a question or resolve a daily-life situation, the response is a cognitive product. The estimation of the quality of this response (confidence in the answer, tendency to use this answer) is of metacognitive nature.” It is now acknowledged that schizophrenic patients present impairments of this skill, to various degrees, which can be assessed by recording ratings of the degree of confidence immediately after the performance of a cognitive task. According to Koren et al. (2006), the extent of the patients’ knowledge of the disease and their involvement in their own treatment are positively correlated with metacognitive skills level. Interestingly, they emphasized a subdivision of the construct into a monitoring component and a control component, respectively, referring to the self-assessment of the performances of a cognitive process, and to the effective use of the results of that process to guide the behavior (for instance, when uncertain, one might not answer in a free-response paradigm).

This short overview of the constructs of major interest for schizophrenia research, brings to light the complex, multifaceted nature of these processes, and consequently of related disorders, undermining the approach of a monodimensional deficit.

### Kaleidoscopic approach of social cognition with batteries of tests

With the unabated successful examples of well-known neuropsychological assessment batteries, several researchers advocated the use of selected sets of social cognitive tests in order to draw a patient’s panorama of skills. It would be obviously out of the scope of the present article to discuss the many attempts to validate a specific set of tests. Of interest is the social cognition psychometric evaluation (SCOPE) initiative from the NIMH, a group of experts who advocated for the use of a social cognitive battery based on separate constructs such as attributional style, emotion processing, ToM, and, eventually, trustworthiness. Nevertheless, they acknowledged that there was no consensus about the constructs to be selected, and a great overlap among both the measures and the domains (Pinkham et al., 2013). It could be argued that this overlap constitutes a key feature of social cognitive processes, which are, by definition, interconnected and mutually informative (see below for discussion of this point). As noted by Keysers and Gazzola (2007), “much of the debate in social cognition might result from choosing tasks that isolate the processes of just one route in the laboratory. However, it is essential to start designing tasks that reflect the complexity of social life to test how the social brain forms an integrated whole.”

There is a considerable experimental evidence to support the content validity of the aforementioned constructs, but their additional contribution to the schizophrenic phenotype remains unclear. First, the relationship of social cognitive disorders with the patients’ complaints is not straightforward. While some authors found extremely limited correlations between ToM deficits and quality of life items (Urbach et al., 2013), other authors found that this potential link was in statistical interaction with symptom severity (Maat et al., 2012). Nevertheless, a much stronger line of evidence emerged during the past years focusing specifically on real-life functioning, outcome, or psychological handicap. Fett et al. (2011) meta-analyzed 42 schizophrenia studies and were able to demonstrate a significant correlation of social cognitive variables with community functioning, which explained around 16% of the latter variable’s variance. Interestingly, these authors concurred with other experts like those of the VALERO project (Leifker et al., 2009), and stressed out the limited consensus about functional outcome constructs and measures. In addition, they noted that neurocognition and social cognition leave a large part of the variance in outcome unexplained.

While causal pathways leading to reduced functioning are multifactorial, we argue that some intermediate psychological constructs merit consideration to explain the variance of schizophrenic patients’ outcome. To solve the puzzle, one has to hypothesize that between the impaired processes that are under scrutiny and negative consequences in real-life situations, some pieces are still missing. First, we suggest that measurements of social cognition could be obtained by testing the domains of impairments cited above in an integrative and ecological manner, rather than separately. However, it is necessary to keep in mind that the technical limitations of real-life ecological assessments by reminding Rus-Calafel et al.’s remark: “observing and practicing the patient’s social skills in natural social interaction/environment could prove useful […] however […] this can be time-costly for the clinician and likely highly intimidating for the patient” (p. 82, 2013). We may add that poor replicability and complex standardization of these procedures will remain a fundamental limitation in obtaining valid dependent variables. Second, recent theoretical accounts brought into the light the specificity of interactive situations in terms of cognitive processes. In the following, we refer to some of these accounts and argue in favor of their relevance to provide ecologically valid insights on some of the putative intermediate links.

## Part II

### Ecological points of views on social cognition

In the late 1980s, Ickes and coworkers suggested the use of the term *naturalistic social cognition* for studies in which the experimental conditions were socially complex situations (Ickes et al., 1986; Ickes and Tooke, 1988). Zaki and Ochsner (2009) recently plead this approach and claimed that, in neuroimaging studies of mental state attribution, researchers ask subjects to make judgments about targets presented as simple stimuli (pictures, cartoons, or texts), but these judgments are generally too easy, because the targets appear to be fictional. They suggested that neuroimaging studies and experimental psychology studies should generate a greater variance in terms of social performance, to prevent floor effects, and should be more ecological and dynamic, more closely resembling real life.

Besides the importance of naturalistic stimuli for the investigation of social cognition, the need for overcoming the spectatorial gap and for adopting a second-person approach has already been proposed by Schilbach et al. (2013). According to these authors, the second-person approach is based on interaction and emotional engagement between people, far beyond a mere observation (p. 395). Thus, constituents of second-person approach are described by the implication of tasks, which require social interaction and emotional engagements. Reciprocity seems also to be a key compound of social interaction and there is a growing literature, which highlights the neurobiological correlates of the reciprocity in social interaction. Social interactions are, according to Schilbach (2014), characterized by “reciprocal relations with the perception of socially relevant information prompting (re-) actions, which are themselves reacted to” (p. 2). The second-person approach allows to investigate not only the way that a persons gathers information about the other person but also one’s knowledge of the other may reside in the interaction dynamics between the agents (Froese et al., 2014).

A successful example of this approach has been realized by Wilms et al. (2010). The authors suggest that the use of gaze contingent stimuli can create a truly interactive paradigm for social cognitive and affective neuroscience. In this work, participants’ own gaze data have been used to animate a virtual character with an interactive eye tracking set-up coupled with a MR scanner. Participants experienced the effects of their own gaze on the virtual agent (for instance, in a joint attention condition, avatar may look to the same object that a subject is looking). This set-up can also be used to investigate neural correlates of one’s being “initiator” or being “responder” in a social interaction. According to authors, the fact that the agent becomes responsive to the participant’s gaze allows the whole set-up to engage and maintain an “online” social interaction.

Given the fact that emotion recognition impairments and gaze abnormalities in social interaction are intricate in psychiatric disorders such as schizophrenia and autism, it should be acknowledged that a second-person approach with naturalistic stimuli in social cognition settings provide specific information (Timmermans and Schilbach, 2014) at the junction of real-life and picture-based emotion recognition assessments. For instance, the experiment proposed by Wilms et al. (2010) is highly relevant to the case of children with autism. As a matter of fact, children with autism show less pronounced impairments in their ability to follow someone’s gaze shifts than in their drive to make someone look at something (Mundy and Newell, 2007).

This is why, according to Zaki and Ochsner, the use of naturalistic social cognition may be critical to understand illnesses involving social cognition deficits. Having examined the particular case of autism spectrum disorders, they concluded, “by moving toward paradigms that capture the complexity of the real social world, and assessing perceivers’ abilities to make accurate inferences about targets, neuroimaging of social cognition can approach more ecologically valid theories about how minds understand each other” (p. 9, 2009). To build upon these theoretical accounts, we identify, in the following, several characteristics in a naturalistic approach.

*First- and second-person perspectives are to be allowed and even privileged*. The mere distant observation of a person as in classical ToM paradigms (pictures, comic-strips, stories, etc.) might disengage shared representations (because they are useless to understand the situation), or neuro-functionally speaking the mirror neuron system, as well as all the processes that allow the immersion into a specific social situation.
*The subject acts upon the stimuli interactively and is not limited to a passive stance*. Methodologically, it is relevant to raise a distinction between a true interaction and a fake interaction. In the former, the reacting subject modifies significantly subsequent stimuli, as in real life. Adaptative model would be required for instance based on personality models (Faur et al., 2013) or complex cognitive architectures (Hemion, 2013). In the latter approach, the subject is presented controlled stimuli that let him believe and act as if he could modify the course of the social events. Graph-structured scenarios with only marginal adaptative capabilities may be used. While, advances in technologies allow both settings, constraints on experimental designs and subsequent statistical analyses often encourage the second solution.
*Interaction with complex social agents leads to a certain degree of unpredictability*. It is conceivable that the social brain is shaped to manage unpredictability although it contributes at making sense of multiple/successive social cues. The experiment from Yoshida et al. (2010) brings intriguing evidence of paracingulate cortex processing uncertainty about another’s agent intentional strategy, i.e., the order of the ToM model used by this agent. Here again, the reality of the interaction is not crucial to elicit the illusions of being immersed into an unpredictable situation, and one might hypothesize that experimental situation with a hidden level of control on the stimuli are sufficient to elicit these neural processes.
*Multimodal stimuli are present most of the time* with a convergence of several channels such as visual perception of social cues (postures, gestures, emotional displays, etc.), auditory perception (verbal utterances, prosody), and eventually haptic components. Notably, the interpretation of the information coming from each channel requires contextualization, integration, and inferences and might involve mental state attribution [this is discussed in Brunet-Gouet et al. (2010)]. As a result, no modality dominates the others and new information has the potential to revise dramatically the current representation. For instance, an incongruous facial emotion at the end of a lengthy discourse may lead to a complete revision of our understanding of the verbal content although the stimulus is quantitatively minimal and qualitatively poorly informative.
*All the inferential processes without exclusion are involved during a naturalistic interaction*, i.e., emotional states inference or empathy, mental state attribution (beliefs, intentions, knowledge, etc.), moral judgment. In other words, naturalistic paradigms should not be structured in such away that disengagement of one process appears relevant to achieve the task.
*Contextualization concerns not only multimodal information but also the succession of events* that make sense when associated within a sequence. While many social cognition paradigms depict an agent performing actions following a specific schema (intentional comic-strips, unexpected transfer tasks, video-based mental state inferences, etc.), many of them do not capitalize sufficiently on a single character in order to promote a real interest in this agent. To enter in a true empathetic relationship, it might be relevant to allow the subject to activate full representation of the other person, including personality traits.

### New methods are provided by researches on virtual reality

The need to investigate social cognition in a more ecological manner will necessitate the use of multimodal, interactive experimental stimuli and to be as respectful as possible to the criteria cited above. Advances in computer technology over the last 20 years made it possible to immerse people in an artificial world and to allow them to interact with the simulated environment. *Virtual reality* is a term commonly used in reference to these technologies that often require realistic, multimodal feedback. Technically, virtual reality requires real-time 3D computer graphics, sound, and other sensory input, and physical models of various degrees of sophistication, to generate a computer-generated environment with which the user interacts (Gregg and Tarrier, 2007). In some cases, haptic, or even olfactory stimuli are proposed [for a review of virtual environments and reality and their application in psychopathology (Baus and Bouchard, 2014), presented in the current research topic]. Recent technological advances offer exciting opportunities to carry out studies of naturalistic cognition, through the use of multimodal stimuli and the extension of the variables measured to physiological and behavioral data. Moreover, the fields of virtual agent simulation and affective computing have matured and now provide credible solutions for interactivity. For these reasons, Kim et al. (2008) argued that virtual reality techniques can overcome traditional assessments’ limitations by composing diverse social situations with various backgrounds and by providing interactive dynamic simulation in social and emotional situations, in opposition of passive observational setups.

Despite the absence of consensus on objectives and methods, psychopathology has also participated to this progress, through the development of new uses of computer graphics, simulation, interface technologies, and social agent modeling such as clinical assessment, intervention, and training. First, new treatments for psychopathological conditions based on virtual reality may prove beneficial (Riva, 2005). According to Rizzo et al. (2012), clinical virtual reality has significant potential to enhance clinical practice and research in three key areas: exposure therapy, neuropsychological assessment, and clinical training with virtual patient agents. Virtual reality settings have been used effectively for the treatment of post-traumatic stress disorder (Rothbaum et al., 2001; Difede and Hoffmann, 2002) and social anxiety (Herbelin et al., 2002; Klinger et al., 2005). These therapies make use of the immersive nature of virtual environments and their ability to trigger emotions corresponding to real-life situations. However, unlike real exposure, the patients are protected against harmful consequences, helping them to accept being confronted with anxiogenic stimulation.

Second, cognitive rehabilitation programs using virtual reality have proved effective for the treatment of patients with unilateral spatial neglect (Kim et al., 2011) and brain injuries (Christiansen et al., 1998). Such programs may include cognitive remediation therapies, during which patients are trained on selected cognitive tasks of increasing processing demand, in order to increase cognitive efficiency and behavioral performances [for a review, see Cicerone et al. (2011)]. Based on simple games, computerized cognitive remediation programs are now frequently offered to patients with psychosis and have been shown to improve cognitive and psychosocial functioning (McGurk et al., 2007b; Wykes et al., 2011).

However, the use of virtual reality in the treatment of schizophrenia has been reported only rarely (Ku et al., 2003; Costa and Carvalho, 2004). Interestingly, the patients participating in the study by Costa and Carvalho agreed to work with computers coupled with immersive glasses and demonstrated a high level of interest in the proposed cognitive tasks. The existence of a specific motivational effect of virtual reality environments may be of clinical importance as motivation is a key factor for a successful remediation therapy (Medalia and Richardson, 2005). Among successful contributions, virtual environments have been used in attempts to improve the social skills (Rus-Calafel et al., 2013) or conversational skills and assertiveness (Park et al., 2011) of schizophrenia patients.

Of importance for the present article, virtual reality can also be used for the evaluation of cognitive impairments in schizophrenic patients. For instance, Ku et al. (2003) used a virtual reality system for the multimodal assessment of cognitive ability in schizophrenia patients. In this work, the task consisted of an adaptation of Wisconsin card sorting test (WCST) to the virtual reality. The program projected through a head mount display presented a pyramid with doors presented like WCST in which schizophrenia patients had to figure how to get out. The main idea here was to use all the benefits of a head mount display for visual and auditory stimulus as well as the haptic feedback (vibration signal for wrong answer). The results showed a correlation of the virtual task and the classical WCST; however, it provided modest information on how patients behaved according to the specific modalities of stimuli. In the same vein, the results obtained by Freeman et al. (2008) suggest that virtual reality environments could be used to assess paranoid thinking in the general population. In this investigation with 200 healthy subjects, the task consisted of a 4 min journey in a virtual London underground projected in a head mounted display. Paranoid thinking as well as other assessments through questionnaires showed significantly that there were a large minority of participants having paranoid thoughts and that this finding was correlated with anxiety, worry, perceptual anomalies, and cognitive inflexibility.

Methodologically speaking, computerized evaluations allow precise measures of behavior with variables such as performance rate, and reactions times, which are classically considered in experimental psychology. A recent study with two virtual agents engaged in a conversation allowed to investigate the gaze patterns of schizophrenic subjects and demonstrated an increased attention to the between-agent space when they spoke, putatively, a form of other’s gaze avoidance (Han et al., 2014). While these authors recorded the patient’s feelings principally in terms of valence during the task, no specific attention was paid to more complex feelings or interpretations. In the following, we will point out that naturalistic experiments based on virtual reality offer diversified means to assess the patient’s strategies to process the task, and to investigate their subjective experiences. Subjective judgments may be recorded both during and after the experiment with specifically designed questionnaires and provide an insight into metacognition and interpretation of controlled social stimuli. Considering the replicability requirements of experimental sciences, we advocate here for the use of such measure to extend our understanding of the patients’ particularities within interpersonal interaction.

Lastly, the choice of realistic simulation techniques to generate social situations and interpersonal interaction raises the issue of the quality of the empathetic relationship that is established with the virtual agent. To a certain extent, the occurrence of empathy may be considered as an extension to the social domain of the broadly speaking “immersion phenomenon” that is the ultimate ambition of virtual environments. *Immersion* is classically defined as the technological power of the system to “deliver an illusion of reality to the senses of a human participant” (Slater and Wilbur, 1997). Immersion could be considered as a precursor leading to a psychological state or even a state of consciousness characterized by perceiving oneself to be enveloped by, included in, and interacting with an environment that provides a continuous stream of stimuli and experiences. As discussed by Witmer and Singer (1998), the conjunction of immersion with a higher *involvement* of the subject (i.e., the “energy” and the attentional resources allocated to the virtual environment) results in the subjective experience of *presence* when the subject privileges the virtual environment over reality. Interestingly, the latter construct was investigated with several anxiety-eliciting virtual situations (TAVE Software) and it was shown that an individual’s anxiety state as well as his/her personality characteristics like introversion influenced positively the sense of presence (Alsina-Jurnet and Gutiérrez-Maldonado, 2010). Moreover, factor analyses of presence self-reports demonstrated the multi-dimensional nature of this construct, distinguishing spatial presence, involvement, realness, and predictability and interaction (Schubert et al., 2001).

Interestingly, the concept of *social presence* was coined to account for the salience of the relationship with an agent, for instance, when using communication tools such as videoconference or avatar-based virtual worlds (i.e., Linden lab’s Second Life) allowing interpersonal interactions, and even extended to the concept of *co-presence*, i.e., the sense of being together (Tugba Bulua, 2012). These different constructs appear to contribute to the subjects’ satisfactory experience, which in turn could improve their motivation. Here, we hypothesize that the building of an empathetic relationship with a virtual agent would constitute a crucial correlate of the experience of his/her presence. As a consequence for clinical researches focusing on the use of virtual agents as a particularly motivating technique, studies should combine assessment of empathy toward the virtual agents, of the subjects’ trait empathetic skills and, more generally of their tendency to immerse. Last, we advocate a better integration of the constructs arising from knowledge on the psychological dispositions related to virtual reality usages, and of those from social neurosciences.

## Part III

### Experimental illustration of investigating schizophrenia with a virtual agent: The virtual card game paradigm

From the many and often heteroclite accounts described above emerges a relevant new empirical approach for exploring the interpersonal disturbances of schizophrenia patients in a more ecological and naturalistic way. This approach focuses on real-time social interaction, requiring the simultaneous integration of all social cognition processes: the attribution of intentions to others, first and second order understanding of mental states, emotion recognition, empathy, metacognition, attributional style, and the contextualization of a given social situation. It would not be reasonable to claim that this approach can test all aspects of social cognition simultaneously. However, this approach makes it possible to study some of these aspects in interaction, and represents a more ecological experimental setting than the batteries of social cognitive tests currently used to assess the impairment of social cognition in schizophrenic patients. In the following, we will show how a precise control of stimuli through computer code specification, allows a simple paradigm to investigate one or several subcomponents thanks to very simple manipulations of the parameters (here, non-verbal communication through emotional displays with varied valence and intensity). Second, we will illustrate the subjective aspects of an immersion into an empathetic relationship through the examination of patients and healthy subjects’ post-experiment reports.

### Description of the virtual card game

**Figure 1:**
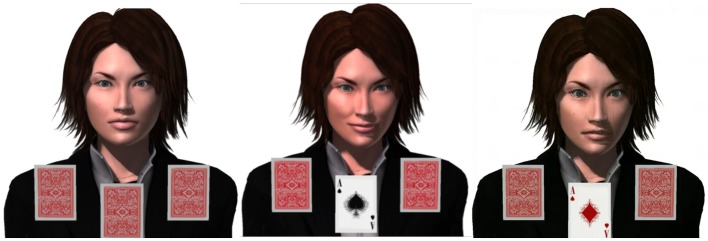
**Screen captures of the experimental setting, showing the affective agent Mary playing a card game**. Left: neutral expression, looking at the subjects. Middle: positive expression, looking at the right card. Right: negative expression, looking at the right card.

The experimental situation was derived from a trust game (i.e., games during which one evaluates anothers’ trustworthiness and intentions in order to produce monetary arbitrages; Berg et al., 1995) from which psycho-economic judgments were removed. The participants were presented a game in which they met a female virtual agent and had to infer from her facial expression displays which card to choose in order to match the color of another card (see Figure 1; Supplementary Material). Of methodological importance, the task is self-explanatory: the agent provides the instructions to be followed, and, at the middle of the game, additional informations are provided to help the subject focusing on emotional displays. However, the participants are not informed before the experiment of the virtual agent’s communicative intentions. Consequently, the attribution of a cooperative intention is left entirely to the subjects’ appreciation.

Technically speaking, the task requires only a multimedia personal computer running the multimodal affective and reactive characters (MARC) framework (Courgeon et al., 2008; Courgeon and Martin, 2009) to animate a realistic 3D character named Mary. MARC is a multicharacter animation platform including real-time body and facial animations based on the computational modeling of emotions (action units models) inspired by the different approaches to emotion in psychology. This platform provides several male and female interactive virtual characters, all of which can speak and simultaneously display subtle facial expressions. In this experiment, we used the female model called Mary, because this model was well liked by participants in preliminary tests of the set-up and had a set of validated emotional expressions (see Supplementary Material).

**Figure 2:**
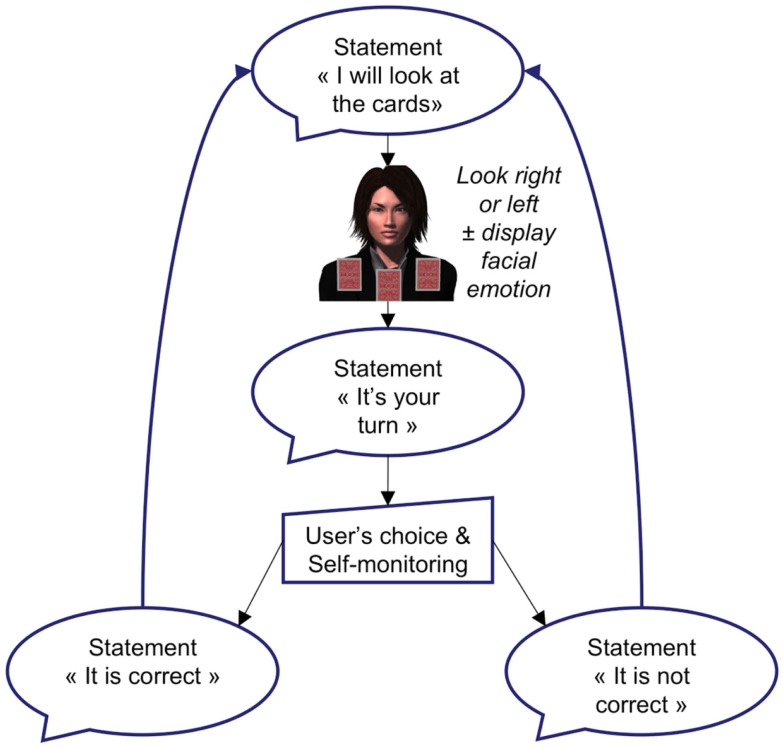
**Procedure: description of a single trial**.

A state machine is used to manage the interaction with subjects following a predetermined scenario (Figure 2). A scenario may be more or less complex and might be seen as an explicit hardcoding of a behavior to simulate a specific social situation. The state machine was responsible for determining the agent’s behavior, i.e., facial expressions to be displayed and verbal statements to be given (if any), as a function of the input provided by the subjects. In the present example, all the state transitions were predefined with the exception of the simulation of small postural movements, blinks, etc. Would the scenario require it, the state transition could be freed to enforce true interaction as defined above. However, it is important noting that the subject was kept blind to the underlying structure of the sequences of events, so that the system generated a fake interaction, which constitutes an intermediate between a true interaction and a classical stimulus/response task.

### How different aspects of social cognition are manipulated within the virtual card game?

As advocated in this article, virtual reality settings based on affective agents constitute a heuristic approach to the needs of naturalistic social cognitive research. We have described above a paradigm, which benefits from real-time 3D rendering of emotions with possibilities to add verbal communication and more or less degrees of freedom in the scenario. In the following, we exemplify how several constructs that are considered of importance in social cognition research are at least represented and, more importantly, can be directly manipulated within the present paradigm.

#### Emotion recognition

Understanding the game and correctly answering requires decoding facial affects, and more precisely, to interpret them as a communication means in the absence of a verbal advice from the agent to choose one card or the other. Although it is quite unusual in other paradigms based on affect recognition, the subjects have to interpret negative emotional displays as helpful cues. Direct manipulation of the emotional displays is easily made as the software platform allows parametric control of action units (valence, intensity, arousal may be defined). In addition, dependant variables concerning emotion are obtained during specific trials in which sympathy ratings on the agent are recorded.

#### Theory of mind

Understanding the helping intention of the avatar is the most fundamental aspect of ToM that is elicited during the game as in any paradigm based on forms of trust judgments. This judgment of cooperation might be easily manipulated by changing the strategy of the agent, and also by changing it adaptively during the game. This would be of major interest to assess patient’s disposition to manage changing relationships and not to perseverate in their own strategy. More subtle is the fact that the current version of the paradigm makes the agent express the correctness of the subject’s answers making it possible to interpret these feedback as incorrect or misleading (i.e., I believe that Mary is lying even when she tells me that my choice is correct). According to a Bayesian framework like in Yoshida et al. (2010) article, such a simple task could bring some insights on the order of ToM model that is used by the subject to perform the task.

#### Metacognition

Measuring metacognitive skills remains technically disputable and somehow artificial. Like Koren et al. (2004) it is quite straightforward to implement measures of both monitoring and control. Self-monitoring could, like in our illustration, be made thanks to the use of self-reports on a trial per trial basis and with the correlations with response times, correct answer rates and, eventually, behavioral correlates (eye scanpaths, gait changes, skin conductance, etc.). Metacognitive control could easily and quite ecologically be implemented by allowing free-choice responses, i.e., non-response.

To conclude from this non-exhaustive argumentary, it appears that simple interactive designs contains enough degrees of freedom to permit simultaneous implementation of several constructs. To go beyond the present attempt to justify construct validity, psychometric studies should be necessary to ascertain the concurrent validity of the numerous dependent variables with regards to more consensual social cognitive tests.

### Insights on empathetic relationship with a virtual agent from subjective reports

First of all, let’s describe the behavioral patterns found while using the experimental paradigm. While healthy subjects were successful at interpreting the emotions displayed by the agent as a form of communication in order to guide their choices (see behavioral data in Supplementary Material), schizophrenic patients were profoundly impaired in this task and exhibited very low performance rates, and increased response time. In our view grounded on the literature, this result is in perfect accordance with the evidences showing that schizophrenic patients have profound impairments in emotion recognition, ToM with slower cognitive processing.

Although the behavioral performance of the patients were low, it was interesting to explore their subjective reports about their encounter with the virtual agent. To do so, participants were asked to complete a short 11-item questionnaire (Table 1). Considering that the evaluation of the sense of presence would not be sufficiently informative on intersubjective aspects, we designed questions focusing specifically on relational empathy and on perception and understanding of the virtual agent’s behavior. Thus, several important aspects are covered by the questions:

1. The realism of the interaction, with questions directly focusing on the fact that the subject behaved with the virtual agent as with a real person (Questions 4–7);
2. The understanding of the behavior and the intentions of the virtual agent, and the nature the mental state that the subject attributed to her (Questions 1, 2, 11);
3. An insight on the subject’s own strategy and on his/her implication (Questions 3, 9);
4. The fact that the subject thought that the virtual agent had an opinion or a feeling about him. These items are of importance for that they would signify a bilateral empathetic experience with a second-person perspective on oneself (Questions 8, 10).

**Table 1:** **Relational empathy questionnaire: mean ratings of agreement expressed as percentages provided by the two populations after they had played with the virtual agent**.

Question	Healthy subjects *N* = 15 (%)	Schizophrenic patients *N* = 14 (%)
Q1. Mary took into account your answers with her reactions	36	45
Q2. Mary changed her attitude sometime in the game	62	64
Q3. You changed strategy during game	68	76
Q4. The game would have been played the same way if you had been in the presence of a real person	68	62
Q5. You looked at Mary’s face as if she was a real person	80	64
Q6. You thought that Mary had her own personality	53	52
Q7. You had the impression of being in the presence of someone during the game	40	52
Q8. You wondered what Mary could possibly think about you	20	29
Q9. You tried to answer correctly in order to be appreciated by Mary	22	17
Q10. Mary did not have a good opinion about you	9	21
Q11. Mary wanted you to succeed in this game	36	44

Participants were asked to indicate the extent to which they agreed with statements concerning their perception of the agent on a four-point Likert scale as follows: completely agree (100%), agree (66.6%), do not really agree (33.3%), and do not agree at all (0%).

The results of the questionnaires indicated that the patients welcomed the use of the affective agent and that they were well motivated for the performance of the task. Their answers were quite comparable to those of healthy participants. Interestingly, participants agreed with the opinion that the virtual card game was realistic and played accordingly. According to Kim et al. (2008), studies with virtual avatars have shown that patients behave as if the virtual avatar was a real person standing in front of them and talking to them. They conclude that an avatar in a virtual environment can be used in interaction studies with schizophrenia patients [see also Ku et al. (2006)].

However, the results presented here draw a more complex picture and may give some orientations for future developments. Questions about the mental states of the agent were mitigated with a tendency to disagree with the fact that the agent took into account their reactions and that she was helpful. Both the patients and the healthy subjects observed that the agent could change her attitude, a fact that is partially true as Mary provides new instructions at mid-game. Last, but of importance, the lower agreement rates concerned Mary’s ability to have an opinion on oneself. This suggests that even if the participants reacted to the agent’s behavior (question 3), they did not forget that Mary was a computer-animated character. Apparently, both healthy participants and patients did not find cues suggesting that the agents had a representation of the participant’s attitude although they inferred a quite negative opinion about them in the agent’s attitude. A first means to address this point and to increase the degree of empathy toward the virtual character consists in making the agent express opinions about the performance of the participant, or ask question about the subjects’ feelings. A second strategy is the introduction of low-level and short-term interaction phenomena. For instance, Timmermans and Schilbach (2014) emphasized the relevance of gaze contingencies and related abnormalities in psychopathological conditions such as schizophrenia, autism, and personality disorders (refer to the article in the present topic). Such technical improvements are compatible with the present platform and could, supposedly, improve the sense of presence elicited by the virtual agent.

## Conclusion and Perspectives

Advances in virtual reality and affective computing not only bring exciting perspectives on experimental methods but also challenge profoundly theories of normal and abnormal social functioning. By allowing replicable experimental designs combining multimodality, contextualized stimuli, interactivity with controlled degrees of unpredictability, as well as intersubjectivity based on first- and second-person perspectives, these techniques urge conceptual innovation. In the present article, we have tried to draw a panorama of some of the emerging concepts that would have to be considered in the future. We illustrated this approach with the presentation of a new paradigm and showed how subjective recording of the patients and healthy subjects experience could help improving the intersubjective phenomena.

We suggest that virtual reality paradigms with affective agents, as presented here, constitute a useful and innovative way of assessing social interactions in patients with schizophrenia. These new techniques could partially overcome the difficulties to predict impaired real-life social functioning, contributing to improve ecological validity of tests. The use of affective and reactive avatars can circumvent the limitations of the batteries of test approach and provide broader information about a patient’s social cognitive skills extending dependent variables to metacognition and subjective experience measures. Indeed, the paradigm presented here makes it possible to investigate globally or separately emotion recognition, mental state attribution, and metacognitive self-evaluation by varying each of these parameters.

As suggested in the experimental illustration, self-reports obtained before, during, and after the experiment might provide additional information about the subjective experience. It would be useful to design and to validate self-report scales to assess the empathetic relationship with a virtual agent. Knowledge on human empathy stressing out the importance of cognitive and affective dimensions could refine the way to measure the components of an empathetic experience beyond the measures of immersion and presence. For that the quality and the vividness of the virtual experience could affect differentially the patient’s motivation depending on his/her own psychopathology and personality, experiments should be conducted to inform designers of virtual reality remediation therapies on the best means to improve these aspects.

## Conflict of Interest Statement

The authors declare that the research was conducted in the absence of any commercial or financial relationships that could be construed as a potential conflict of interest.

## Supplementary Material

The Supplementary Material for this article can be found online at http://www.frontiersin.org/Journal/10.3389/fnhum.2015.00133/abstract
