# Supplementary material for: How and Why Affective and Reactive Virtual Agents Will Bring New Insights on Social Cognitive Disorders in Schizophrenia? An Illustration with a Virtual Card Game Paradigm
Source: Front Hum Neurosci. 2015 Mar 30;9:133. doi: 10.3389/fnhum.2015.00133 (PMC4378306; doi:10.3389/fnhum.2015.00133)
Supplement: Supplementary file 1 [file Presentation_1.PDF]

Supplementary material

Source article : How and why affective and reactive virtual agents will bring new insights on social cognitive disorders in schizophrenia? An illustration with a virtual card game paradigm. Oker, A. Prigent, E. Courgeon, M. Eyharabide, V. Urbach, M. Bazin, N. Amorim, M-A. Passerieux,C. Martin, J-C. & Brunet-Gouet, E.

Description of the participants

We tested 14 schizophrenic patients and a group of 15 healthy subjects with preserved social cognitive performances (i.e. students, care-givers, etc.). The healthy subjects had no history of psychiatric follow-up or of psychotropic medication use. All the patients included in this experiment had been diagnosed with schizophrenia according to DSM-IV criteria and were taking antipsychotic medication. The symptoms of the patients were assessed with the positive and negative syndrome scale (PANNS, Kay *et al.*, 1987). All patients had normal or corrected-to-normal vision. The subjects were right-handed, according the Edinburgh handedness inventory, native French speakers and they had no neurological illness. All participants gave their informed consent for participation in the protocol, which was approved by the local ethics committee. The mean age and sex ratio of all participants and the PANNS scale scores of the patients are presented in table suppl. 1. A facial expression recognition test was administered (TREF, Gaudelus & Franck, 2012). This forced-choice test is based on six basic universal emotions: anger, disgust, contempt, happiness, sadness and fear. The score for each participant was calculated as the percentage correct responses for the identification of facial emotions.

Description of the virtual card game

In the current version, the virtual card game requires a microcomputer (Intel([R]) Core(™)i7-3630QM CPU @ 2.40 GHz with 8 Go RAM and NVIDIA Quadro K4000M GPU @ 600 MHz with 4 Go RAM) with a 22" screen and a resolution of 1920 x 1080 pixels, displayed with a refresh rate of 60 Hz. The position of the chair is fixed such that the participants center their gaze on the middle of the screen and the distance between the participant and the monitor is about 55 cm. This setting provides horizontal and vertical visual angles 47 degrees and 27 degrees, respectively. The face of the 3D character appears in the center of the screen, corresponding to horizontal and vertical visual angles of 13 degrees and 10.8 degrees, respectively. The virtual agent was able to produce verbal messages by non-prosodic speech synthesis (Virginie Voice, Scansoft Inc.). The speech rate was kept at the default setting for all participants. At any step of a trial, spoken messages were selected pseudorandomly from several synonymous utterances to avoid unnatural repetitions.

Facial expressions were based on the Facial Action Coding System – FACS (Ekman and Friesen, 1978; Ekman, 2002). FACS is one of the few models capable of describing and classifying the characteristics of human facial expressions. These expressions are characterized in terms of the action units (AUs) activated, with each AU corresponding to the contraction of one or several facial muscles. In this study, the expression of joy used for positive emotional displays involved three AUs: AU6, raising the cheeks (orbicularis oculi, pars orbitalis contraction), AU11, deepening the nasolabial crease (zygomaticus minor contraction), and AU12, pulling up the corners of the lips (zygomaticus major contraction). Expressions with a negative valence were based on fear, which involved four AUs: AU1, which raised the inner brow (frontalis, pars medialis contraction), AU2, raising the outer

brow (frontalis, pars lateralis contraction), AU5, raising the upper eyelid (levator palpebrae superioris contraction), and AU26, which caused the jaw to drop (masseter contraction).

The positive and negative emotions defined above were validated before the experiment. This initial testing was carried out with the same MARC software framework (Courgeon *et al*., 2008; Courgeon and Martin, 2009). Twenty healthy volunteers were asked to identify the expressions of the virtual agent and to indicate whether they found the expression positive or negative. One subgroup made these judgments within the context of the card game (see below for a description of the experimental trials), whereas the others made their judgments outside of the game context (by watching the avatar's expressions in sequential movie clips). Without the card game context, 89.9% of participants identified the expression of joy as a positive expression, and 91.7% of participants also identified the fear expression as negative. In the context of the game, these proportions were lower, at 87.5% and 78%, respectively. However, the difference between the results obtained with and without the game context was not significant ($F<1$).

Virtual card game procedure

The subjects are told that they would be taking part in an experiment in which they play a game of chance, with no monetary gain or loss, with a 3D character. The subjects are also told that the agent will explain the rules and the procedure of the game. To prevent or reduce a potential social presence effect, the experimenter may stay in the room, but he or she remains silent and seated at another desk, looking at neither the subject nor the computer screen. The agent faced the participant, with three cards turned toward her. The agent turned over the card in the middle, explaining what she was doing. She explained that

the task was to guess which of the remaining cards was of the same color (black or red), using the keyboard to select the card on the left or the card on the right, as appropriate. The game began with eight training trials that were excluded from analyses. Every trial started with a verbal announcement (e.g. “let’s go”). One second later, the middle card, which could be either red or black, was shown. After another second, Mary said that she would look at one of the other cards. The direction of head and gaze movement was randomized, with a balance between the numbers of movements to the left and to the right. Card color was also randomized, ensuring similar numbers of red and black cards. Once aware of the card’s color, Mary turned her head towards the card to express either a positive emotion if this card was correctly matched, a negative emotion if this card was not matched or a neutral emotional expression. The winning rate was equilibrated at 50% for the neutral expression. For non-neutral expressions, a low intensity (intensity <50% that for high intensity) or high intensity of expression was selected at random, with counterbalancing to ensure similar frequencies for the two intensities. Once the agent had looked at the card for 0.6 s, she urged the participant to choose a card within the next 8 s. If the subjects failed to respond within the allotted time, the agent then repeated that it was their turn to play. Immediately after their had answered, the participants were asked to provide a metacognitive judgment and rated the accuracy of their judgments on a six-point Likert scale (0%, 20%, 40%, 60%, 80%, 100%; 0% indicated that they were not sure about their choices, whereas 100% corresponded to absolute certainty). At the end of the trial, the agent provided verbal feedback without emotional display indicating whether the participant’s choice was correct or incorrect.

Overall structure of a virtual card game session

Please, refer to Figure suppl. 1. After the initial instructions and training, the trials were organized into blocks including one of each type, by varying valence and intensity (i.e. 8 trials per block). Trial orders were randomized within a block. In the middle of the experiment, the virtual agent provided additional advice: “Attention, new instructions should now be followed. You should carefully look at the expression on my face. Whenever I look at the cards, you should look at the emotions I express. Try to follow these instructions until the end of the game.” After these instructions had been issued, three blocks of the game were carried out. At the end of the third block of games in explicit conditions, every participants were asked to complete subjective experience questionnaires after each trial for a series of six turns of the game which cover every experimental condition. Two questions were asked: 1. To what extent did Mary help you? 2. To what extent did you find Mary sympathetic? Subjects were asked to provide a response to these questions on a six-point Likert scale (0%, 20%, 40%, 60%, 80%, 100%; 0% indicated that Mary provided no help or was not sympathetic; 100% indicated that Mary helped them as much as possible and that she was extremely sympathetic). At the end of these blocks, relational empathy questionnaires were administered to the subjects.

References

Courgeon, M., and Martin, J.-C. (2009). Impact of Expressive Wrinkles on Perception of a Virtual Character's Facial Expressions of Emotions. *IVA.*

Courgeon, M., Martin, J.-C., and Jacquemin, C. (2008). MARC: a Multimodal Affective and Reactive Character. *1st Workshop on Affective Interaction in Natural Environments*, 12-16.

Ekman, P. (2002). *Facial action coding system*. Salt Lake City: A Human Face.

Ekman, P., and Friesen, W V. (1978). *The facial action coding system*. Palo Alto, CA: Consulting Psychologists Press.

Gaudelus, B. and Franck, N., (2012). *Troubles du traitement des informations faciales. Le programme GAÏA*. In N., Franck, Remédiation cognitive, Paris, Elsevier-Masson.

Kay, S. R., Fisz-Bein, A., and Opler, L. A. (1987). The positive and negative syndrome scale (PANSS) for schizophrenia. *Schizophrenia Bulletin,* 13, 261-274.

	Healthy subjects<br><br>N=15	Schizophrenic patients<br><br>N=14
Males / Females	4 / 11	12 / 2
Age (SD)	29 (13)	37 (12)
PANNS Negative (SD)		18.9 (5.7)
PANNS Positive (SD)		12.9 (5.1)
PANNS Total (SD)		65.9 (18.5)
TREF Total Score (SD)	72.2% (9.9)	55.1% (10.8)

Table suppl. 1. Mean age, sex ratio of all participants, PANNS scales of patients and standard errors (in parentheses).

	Healthy subjects<br><br>N=15		Schizophrenic patients<br><br>N=14	
	**Ratings or performances**	**Response time (ms)**	**Ratings or performances**	**Response time (ms)**
**Behavioral performances**				
Positive expression	80%	1633	63%	2174
Negative expression	54%	1802	42%	2138
Neutral expression, zero-intensity	Not applicable	1957	Not applicable	2270
Low intensity expression	70%	1719	49%	2009
High intensity expression	64%	1718	56%	2309
**Metacognition**				
Metacognition Self-monitoring degree<br><br>(within each trial)	70%		62%	
Metacognition Helpfulness<br><br>(last block only)	54%		50%	
Sympathy<br><br>(last block only)	50%		55%	

Table suppl. 2. Performances and reaction times during the virtual card game. Metacognition ratings.

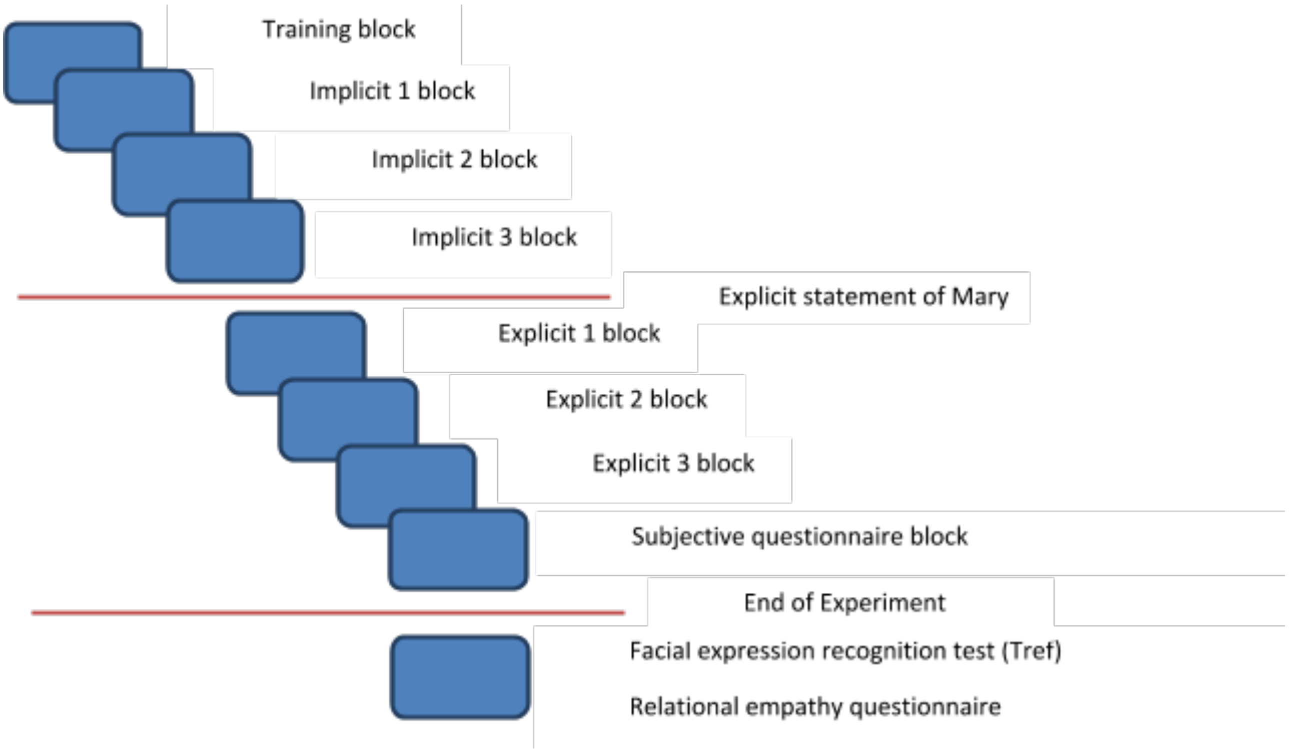

Figure suppl. 1. Flow chart showing the order of the experimental blocks.
